# Systematic genome-wide and expression analysis of RNA-directed DNA methylation pathway genes in grapes predicts their involvement in multiple biological processes

**DOI:** 10.3389/fpls.2022.1089392

**Published:** 2022-12-09

**Authors:** Rui Xiang, Bilal Ahmad, Chen Liang, Xiaoxin Shi, Lili Yang, Guoqiang Du, Li Wang

**Affiliations:** ^1^ College of Horticulture, Hebei Agricultural University, Baoding, China; ^2^ Department of Horticulture, Muhammad Nawaz Sharif (MNS)-University of Agriculture Multan, Multan, Pakistan; ^3^ Shijiazhuang Fruit Research Institute, Hebei Academy of Agricultural and Forestry Sciences, Shijiazhuang, China

**Keywords:** RdDM, seed development, grapes, DNA methylation, expression analysis

## Abstract

RNA-directed DNA methylation (RdDM) is an important epigenetic pathway in plants and mediates transcriptional silencing by siRNAs. Different gene families have role in the regulation of the RdDM pathway and there is a lack of information about these gene families in the grapes (*Vitis vinifera* L.). Here, we mentioned the genome-wide identification, bioinformatics analysis, evolutionary history, and expression profiling of *VvRdDM* pathway genes against various stresses, hormonal treatments as well as in different organs. Sixty *VvRdDM* genes belonging to fourteen different families were identified. All the genes were unevenly distributed and chromosome 4 contained the highest number of genes (7). Most of the genes showed similar exon-intron and motif distribution patterns within the same subfamilies. Out of 14 families, only members of 4 families underwent duplication events during the evolutionary process and 50% of members of the AGO family are the result of duplication events. Based on Ka/Ks ratio all duplicated gene pairs have a negative mode of selection. *VvRdDM* pathway genes showed differential spatiotemporal expression patterns against different hormone and stress treatments. Further, with multiple transcriptome analysis, some *VvRdDM* genes showed a broad spectrum of high expression in different organs at various stages, and *VvRdDM* genes also displayed different expression in seeded and seedless cultivars during different phases of seed development. This proposed that *VvRdDM* genes may play multiple roles in grape growth and development, especially in seed development. qRT-PCR analysis of selected genes further verified the critical roles of RdDM genes in multiple biological processes, especially in seed development/ovule abortion i.e., *VvIDN2a*, *VvDRD1a*, *VvRDR1a*, and *VvRDR6*. Our study provides detailed information about *VvRdDM* genes in perspective of gene structure and evolution, as well as expression pattern against different stress, hormones and in different plants parts. It provides new candidate gene resources for further functional characterization and molecular breeding of grapes.

## Introduction

Epigenetics investigates heritable changes in gene function that occur without a change in DNA sequence (Holliday, 2006). Most of the epigenetic changes (Histone modification, DNA methylation, chromatin remodeling, and chemical modification) occur due to microRNAs (miRNAs). The epigenetic mechanisms are tightly connected to cellular development and differentiation (Barter et al., 2012). DNA methylation is a conserved epigenetic silencing process that is involved in a variety of biological functions, including the regulation of gene expression, prevention of the spread of transposons, and defense against these organisms. Additionally, changes in DNA methylation can result in abnormal development (Zhang et al., 2018). Mostly DNA methylation in plants occurs in the CG, CHG, and CHH sequences (where “H” can be either an A, C, or T), and it is especially prevalent over heterochromatic transposable elements (TEs) and repetitions, where it is crucial for transcriptional gene silencing (TGS) (Zhang et al., 2018). However, many plant species have DNA methylation over gene bodies but in this case precise role is still unknown (Bewick and Schmitz, 2017).

In plants, DNA methylation usually happens by RNA-directed DNA methylation (RdDM). RdDM is a biological mechanism in which DNA methylation is controlled by non-coding RNA molecules. RdDM-induced DNA methylation is usually linked to transcriptional suppression of the genomic regions targeted by the pathway (Erdmann and Picard, 2020). DNA methylation can change gene expression through direct and indirect mechanisms. Further, when it occurs in promoter region of a gene it inhibits transcription (Wierzbicki et al., 2008; Zhang et al., 2018). The key players of RdDM pathway are Pol IV (RNA Polymerase 4), RDR2 (RNA-dependent RNA polymerase 2), DCL3 (Dicer-like 3), and AGO4 (Argonaute 4) (Dinh et al., 2013). According to the canonical RdDM pathway, which is best explained in Arabidopsis, generation of 24-nt small interfering RNAs (siRNAs) depends on Pol IV. Pol IV transcribes single strand RNA (ssRNA), which is converted to double strand RNA (dsRNA) by RDR2. Then dsRNA is diced by protein DCL3 resulting in siRNAs (siRNAs 24nt). These siRNAs are loaded in AGO4 protein and AGO4 interacts with RISC (RNA-induced silencing complex) and methylates DNA (Matzke and Mosher, 2014). Apart from these proteins, some other proteins also play roles in RdDM pathways for example, SHH1 (SAWADEE HOMEO-DOMAIN HOMOLOG 1), CLSY (CLASSY), NRPD (NUCLEAR RNA POLYMERASE D), DRM (DOMAINS REARRANGED METHYLASE), RDM (RNA- DIRECTED DNA METHYLATION), RRP6L (RRP6-like), DRD (DEFECTIVE IN RNA-DIRECTED DNA METHYLATION), DDM (DECREASE IN DNA METHYLATION), IDN (INVOLVED IN DE NOVO), HEN (HUA ENHANCER). Moreover, SHH, NRPD, NRPE (DNA-DIRECIED RNA polymerase V subunit 1), DRM, RDM1, RDM2, RDM3, RMD4, and RDR have SAWADEE, RNA_pol Rpb2, RNA_pol Rpb1, DNA methylase, RdDM_RDM1, RNA_pol Rpb4, Spt5-NGN, lwr1, and RdRP conserved domain, respectively (Wassenegger and Krczal, 2006; Ream et al., 2009; He et al., 2009a; He et al., 2009b; Henderson et al., 2010; Hirtreiter et al., 2010; Zhang et al., 2013; Sasaki et al., 2014). CLSY, DRD, and DDM have two conserved domains; Helicase_C and SNF2-rel_dom (Smith et al., 2007; Hu et al., 2013). RRP6L also has two domains (DNA_pol_A_exo1 and HRDC) (Lange et al., 2008). IDNs (zf-XS, XS and XH), HEN (Hen1_Lam_C, Methyltransf_31, and dsRBD2), and AGO (n-side PAZs, C-side Piwi, and ArgoMid) each has three conserved domains (Ausin et al., 2009; Huang et al., 2009; Zhao et al., 2014). Further, DCL has six conserved domains (DEAD, Helicase-C, Dicer_dimer, PAZ, Ribonuclease_3, and dsrm) (Margis et al., 2006). In most of the crops, AGO, DCL, and RDR gene families have been identified and characterized.

DNA methylation performs diverse functions in plant growth and development including stress tolerance (Zhang et al., 2018). RdDM-related genes have been well studied in various crops for their critical roles in stress tolerance. For example, RNA Pol V (NRPE), *RDR1/2/6*, *DCL2/4*, *CaDCLs*, and *RDR1* have been reported for their roles against different stresses in different crops i.e., Arabidopsis and pepper (Garcia-Ruiz et al., 2010; López et al., 2011; Qin et al., 2018). Further, *SlAGO4* showed a negative role in salt and drought stress tolerance in tomato (Huang et al., 2016). DNA methylation may have potential roles in flowering, fruit setting, seed development, and fruit ripening. In Arabidopsis low levels of methylation caused delay in flowering (Jones and Sung, 2014). *AtRRP6L1* and *AtRRP6L2* regulated flowering in Arabidopsis *via* repressing the expression of *FLC* (*Flowering Locus C*) (Shin and Chekanova, 2014). On the other hand, high levels of DNA methylation are important for orange fruit development and ripening (Huang et al., 2019). In the abscission zone of the citrus sinensis fruit, key players of the RdDM pathway are down-regulated (Sabbione et al., 2019). Further, low DNA methylation levels in apples resulted in reduced fruit size (Daccord et al., 2017). In tomato a change in hypermethylation in *CNR* (*COLORLESS NON-RIPENING*) indicated abnormal fruit ripening (Manning et al., 2006). Cheng et al. (2018) has also reported the involvement of RdDM pathway in strawberry fruit ripening. During the ripening process in apples, DNA methylation of the *MdMYB10* promoter affected gene expression and fruit color (El-Sharkawy et al., 2015).

The role of RdDM in seed development and germination has been studied (Xiao et al., 2006; Zhong et al., 2020). Furthermore, the data suggested that DNA methylation can play a critical role in seed dormancy (Kawakatsu et al., 2017). The association of DNA methylation with seed development has been reported in *Arabidopsis thaliana* and *Brassica rapa* (Grover et al., 2018). For example, the involvement of *AtHEN1* in ovule development (Wei et al., 2020). Various phases of tissue have different degrees of DNA methylation. For example, the shoot apical meristem of early peach seedlings exhibited a higher amount of DNA methylation than adults (Bitonti et al., 2002). Most probably, the diverse DNA methylation levels in different tissues at different growth stages are due to different DNA methylation pathways (Bartels et al., 2018) and gene expression levels.

Grapevine (*Vitis vinifera* L.) is among the top fruit crops grown all over the world. The grapes are consumed in different ways including fresh fruit (table), wine, juices, and raisins (dried) (This et al., 2006). RdDM pathway genes have been identified in different plant species including Arabidopsis (Kurihara et al., 2008), *Brassica rapa* (Cao et al., 2016), Soybean (An et al., 2017), Citrus (Mosharaf et al., 2020), Apple (Li et al., 2019), Rice (Ahmad et al., 2019), Maize (Qian et al., 2011), Tomato (Bai et al., 2012), and Ginkgo (Gao et al., 2020). However, in most of the species scientists have reported only three RdDM (DCL, AGO, and RDR) gene families. There is a lack of information about *RdDM* genes in grapevine. The involvement of *RdDM* genes in different mechanisms of plant growth, development, and response to different stresses (biotic and abiotic) and antiviral defense, justifies the need of detailed bioinformatics studies of *RdDM* genes in grapevine. Here, we elaborated comprehensive genome-wide identification of *RdDM* grapevine genes, including phylogenetic analysis, chromosomal positions, intron-exon distribution, motif analysis, evolutionary history, selection pressure, and cis-elements analysis. Various transcriptome analyses under biotic stress, abiotic stress, hormone treatments, and at different stages of growth were performed. The expression of selected genes was further investigated during progressive phases of seed development in seedless and seeded cultivars. Moreover, the integration of all these studies can provide inklings about *VvRdDM* pathway genes function and their involvement in multiple biological processes. Our study provides basic information about *RdDM* grapevine genes and will provide new candidate genes for functional studies.

## Materials and methods

### Identification of RNA-directed DNA methylation genes in grapes

To identify RdDM pathway genes in grapes, the grape genome was retrieved from Grape Genome CRIBI Biotechnology (http://genomes.cribi.unipd.it/) and Grape Genome Database (http://www.genoscope.cn.fr). Protein sequences involved in the RdDM pathway of the Arabidopsis were downloaded from the TAIR database (http://www.arabidopsis.org/) and were used as a query in Pfam database (http://pfam.xfam.org/search#tabview=tab1) to obtained the Hidden Markov Model (HMM) profile of the RdDM conserved domain. SPDE software was used with the hmmer search function (HMMER 3.0) to obtain candidate genes (Xu et al., 2021). Arabidopsis RdDM pathway protein sequences were used as a query in National Center for Biotechnology Information (NCBI; http://www.ncbi.nlm.nih.gov/), Grape Genome Database, and Grape Genome CRIBI Biotechnology using BLAST-P program (e-value threshold of 1e^−10^) (Altschul et al., 1997) to search for sequences of *VvRdDM* homologous genes. The genes obtained from HMM and BLAST-P were combined. To further determine the reliability of RdDM pathways genes in grape, search results of the protein sequences were checked with SMART (http://smart.embl-heidelberg.de), Pfam database and proteins with incomplete domains were removed.

### Cis-acting regulatory elements prediction

2 Kb upstream region (Xi et al., 2017) of the *VvRdDM* genes was retrieved from the latest *Vitis vinifera* genome assembly and annotations were downloaded from Phytozomev12 (http://www.phytozome.net) using the SPDE. PlantCARE database was used for conserved cis-elements prediction (Lescot et al., 2002).

### Phylogenetic analysis and nomenclature

The full length amino acid sequences of RdDM proteins belonging to *A. thaliana*, *Oryza sativa*, *Solanum lycopersicum*, and *V. vinifera* were downloaded from TAIR and JGI Data Portal (https://data.jgi.doe.gov/). Multiple alignment of RdDM protein sequences was performed using ClustalW program with default settings. MEGA7.0 software was used for phylogenetic tree construction (Kumar et al., 2018). The phylogenetic trees were generated with the following parameters; Neighbor-Joining (NJ) method, p-distance, complete deletion, and 1000 bootstrap replicates (Ahmad et al., 2020). The candidate grapevine genes were named based on phylogenetic relationships and sequence homologies with corresponding Arabidopsis homologs (Grimplet et al., 2014).

### Exon-intron distribution and conserved motifs analysis

The conserved motif distribution patterns of *VvRdDM* genes were assessed using Expectation Maximization for Motif Elicitation (MEME) online software (version 5.4.1) (https://meme-suite.org/meme/tools/meme) (Bailey and Elkan, 1994) with the following parameters: default settings and maximum number of motifs were designated to identify 20 motifs. SMART program and Pfam database were used to annotate the MEME motifs. TBtools were used to visualize the results of conserved motif analysis (Chen et al., 2020). The full genomic sequences and respective coding sequences of RdDM grape genes were obtained from Grape Genome Database and online Gene Structure Display Server 2.0 (http://gsds.gao-lab.org/index.php) was used for exon-intron analysis.

### Evolutionary history analysis and estimation of selection pressure

Tandem duplication was determined using the Houlb-described criterion (Holub, 2001). The Plant Genome Duplication Database (http://chibba.agtec.uga.edu/duplication/) was used to retrieve syntenic pairs between Arabidopsis and grapes and among grape genes (Lee et al., 2012). The Circos diagrams were created using TBtools (Chen et al., 2020). The synonymous substitution rate (Ks) and non-synonymous substitution rate (Ka) of duplicated genes were calculated using an online software (http://services.cbu.uib.no/tools/kaks). For estimating selection pressure, the Ka/Ks ratio was calculated (Siltberg and Liberles, 2002).

### Plant materials

In this experiment, 2 seeded (‘Zuijinxiang’ and ‘Kyoho’) and 2 seedless (‘Crimson seedless’ and ‘Flame seedless’) cultivars were used. All the plants were grown in grapevine germplasm orchard of Hebei Agricultural University, Baoding, Hebei, China (38°51′N, 115°29′E) under field conditions, where the average annual temperature is 13.4°C, (Below - 4.3°C in winter and above 26.4°C in summer), the average annual sunshine hours are 2511, and the mean annual precipitation is 498.9 mm. Seed samples were taken from berries using tweezers at 4 different growth stages, 20, 30, 40, and 50 days after full bloom (DAF). All the samples were quickly dipped in liquid nitrogen and preserved at -80°C for further studies. Three biological replicates were obtained at each time point.

### Expression analysis of RdDM pathway genes in grapes

Expression analysis of the *VvRdDM* pathway genes were carried out using transcriptome data of NCBI SRA database (https://www.ncbi.nlm.nih.gov/sra/?term=), the corresponding accession IDs are as followings: for biotic stress treatment: downy mildew infection (SRP013835), botrytis cinerea infection (SRP120480), powdery mildew infection (SRP253455); For abiotic stress treatment: cold (SRP202053), heat (SRP091989); For hormone treatment: gibberellic acid (GA_3_) (SRP045605), methyl jasmonate (MeJA) and salicylic acid (SA) (SRP378285), abscisic acid (ABA) (SRP098802); For organ development at different stages: seeds (SRP081137), buds (SRP159132), inflorescences (SRP045605), berries (SRP265116). Then the raw transcriptomic data was checked using FastQC (v0.11.9) software, and low-quality reads and adapters were removed using Trimmomatic v0.39. All clean reads were mapped to the *V. vinifera* reference genome (PN40024.v4) by Hisat2 software. Gene expression quantification [Transcripts Per kilobase of exon model per Million mapped reads (TPM)] were estimated using StringTie (v2.2.1) (Pertea et al., 2016). R package DeSeq2 (Love et al., 2014) with the principle adjusted p-value (padj) < 0.05 and the |log2 fold change (log2 FC) | ≥ 1 was used for the identification of significantly differently expressed genes (DEGs). Heat maps were generated using TBtools.

### RNA extraction and qRT-expression profiling

The OmniPlant RNA Kit (Dnase I, Comwin Biotech, Beijing, China) was utilized for the extraction of total RNA. For checking the quality and OD value, Agarose gel electrophoresis and nano drop spectrophotometer (Thermo Fisher Scientific, Yokohama, Japan) were used, respectively. Reverse transcription was carried out after gDNA was eliminated using the Prime ScriptRTase (Trans Gen Biotech, Beijing, China). gDNA Eraser (contains Dnase) eliminated genome DNA. Additionally, a qPCR assay on crude RNA was carried out to determine the level of gDNA contamination. Then cDNA was diluted to 200 ng/μl by using distilled water. Primer Premier 7 software was used for designing specific primers (**Supplementary Table S1**). Each reaction was carried out in 20 μl volume comprising of 1 μl cDNA,1 μl of each primer (1.0 μM), 10 μl of 2 X Fast Super EvaGreen^®^ qPCR Mastermix (US Everbright Inc., Suzhou, China), and 7 μl sterile distilled H_2_O. Every reaction was performed three times. With three biological and three technical replicates, the quantitative real time-PCR (qRT-PCR) was carried out using the SYBR Green (Applied Biosystem) in the LightCycler^®^96 System (Applied Biosystems, Roche, Shanghai, China). We used the grape *ACTIN* gene (GenBank Accession number NC 012010) as an internal reference, and relative gene expression was computed using the 2 ^−ΔΔCT^ method (Livak and Schmittgen, 2001).

### Statistical analysis

SPSS Statistics 22.0 software was used for data analysis. One way analysis of variance (ANOVA) on ranks followed by tukey *post-hoc* analysis was used to evaluate the overall significance of the data (Tukey’s test; *p* < 0.05) (Wang et al., 2020). The graphs were created using sigma Plot 14.

## Results

### *VvRdDM* genes identification

There were 60 RdDM genes found in grapes. The obtained genes were structurally analyzed and all genes were divided into different gene families according to conserved domains. There were 4, 2, 3, 2, 1, 9, 2, 10, 1, 3, 1, 4, 5, and 13 genes in SHH, CLSY, NRPD, NRPE, DRM, RDM, RRP6L, DRD, DDM, IDN, HEN, DCL, RDR, AGO sub-family, respectively. The protein prediction lengths of SHH, RDM, DRD, DCL, RDR, and AGO sub-families ranged from 248- 697, 174-1034, 729-2266, 1340-1689, 919-1128, and 879-1039 amino acids, respectively. The complete information about *VvRdDM* genes including gene locus ID, chromosomal position, coding sequence length, and protein sequence length are shown in **Table 1**. All the genes were named as per earlier criteria i.e., based on protein sequence similarity with Arabidopsis RdDM genes.

**Table 1:** List of *VvRdDM* genes.

Gene Name	Accession No.	VCost.v3 ID	Gene Locus ID	Chr.	Start	End	CDS (bp)	ORF (aa)
*VvSHH1a*	XP_002283948.1	Vitvi09g00305	GSVIVT01017001001	9	3362926	3368211	744	248
*VvSHH1b*	XP_002277697.2	Vitvi04g00007	GSVIVT01035290001	4	81666	94722	738	246
*VvSHH2a*	XP_010644158.1	Vitvi19g00157	GSVIVT01014258001	19	2065687	2074580	1260	419
*VvSHH2b*	XP_010654302.1	Vitvi08g02188	GSVIVT01034198001	8	14493878	14509826	2082	694
*VvCLSY1*	XP_019081447.1	Vitvi02g00600	GSVIVT01013277001	2	5723377	5725314	1665	555
*VvCLSY2*	XP_010658217.1	Vitvi13g00115	GSVIVT01032746001	13	1072521	1084172	4908	1635
*VvNRPD1*	XP_010661365.1	Vitvi02g00414	GSVIVT01019869001	2	4035665	4081274	4080	1359
*VvNRPD2a*	XP_002283296.1	Vitvi04g01927	GSVIVT01035963001	4	6537828	6551330	3663	1221
*VvNRPD2b*	XP_002274051.1	Vitvi12g00313	GSVIVT01020543001	12	4643379	4650715	3582	1193
*VvNRPE1*	XP_002265533.1	Vitvi13g01420	GSVIVT01013491001	13	1672655	1730512	5673	1891
*VvNRPE3B*	XP_002285322.1	Vitvi03g00543	GSVIVT01003174001	3	5981436	5989264	621	206
*VvDRM1*	XP_019079260.1	Vitvi12g02119	GSVIVT01023152001	12	21903254	21909960	1383	460
*VvRDM1*	XP_019075614.1	Vitvi05g00537	GSVIVT01018017001	5	5505841	5509295	525	174
*VvRDM2a*	XP_010646234.1	Vitvi10g01687	GSVIVT01003467001	Un	10539130	10543945	720	240
*VvRDM2b*	XP_002282413.1	Vitvi12g02216	GSVIVT01020782001	12	2049955	2053435	417	139
*VvRDM3a*	CBI24807.3	Vitvi13g01507	GSVIVT01000315001	Un	2999773	3004686	684	227
*VvRDM3b*	XP_010658348.1	Vitvi13g00674	GSVIVT01016203001	13	6641768	6659876	2028	676
*VvRDM3c*	CBI33588.3	Vitvi14g01042	GSVIVT01030928001	14	19211208	19212047	375	125
*VvRDM3d*	RVW12274.1	Vitvi19g01303	GSVIVT01038034001	19	16557000	16569957	756	252
*VvRDM3e*	XP_002265283.2	Vitvi18g02832	GSVIVT01010022001	18	13526074	13535704	3105	1034
*VvRDM4*	XP_010650840.1	Vitvi06g01773	GSVIVT01024608001	6	8458427	8471269	1068	355
*VvRRP6L1a*	XP_002269553.2	Vitvi08g00120	GSVIVT01029953001	8	2060775	2111425	2706	901
*VvRRP6L1b*	XP_010657201.1	Vitvi12g00367	GSVIVT01030425001	12	5464697	5472560	2808	936
*VvDRD1a*	XP_002273814.2	Vitvi03g00003	GSVIVT01024225001	3	24426	40615	2571	876
*VvDRD1b*	XP_010660172.1	Vitvi14g00743	GSVIVT01023393001	14	12730208	12834845	4434	1477
*VvDRD1c*	XP_010656983.1	Vitvi12g00289	GSVIVT01020568001	12	4227744	4234100	2625	874
*VvDRD1d*	CBI37137.3	Vitvi07g00624	GSVIVT01028359001	7	6562398	6570091	3351	1117
*VvDRD1e*	XP_002264260.1	Vitvi04g01734	GSVIVT01026450001	4	23417241	23454255	3132	1044
*VvDRD1f*	XP_010653739.1	Vitvi08g01952	GSVIVT01033231001	8	22355168	22372711	4674	1557
*VvDRD1g*	XP_003631348.1	Vitvi01g01488	GSVIVT01010282001	1	18955322	18986890	2187	729
*VvDRD1h*	XP_002275100.1	Vitvi06g01262	GSVIVT01037235001	6	17025273	17066889	4620	1540
*VvDRD1i*	XP_010649878.1	Vitvi05g00453	GSVIVT01017921001	5	4705770	4728522	3333	1110
*VvDRD1j*	XP_010649796.1	Vitvi05g01863	GSVIVT01017820001	5	3724867	3754247	6801	2266
*VvDDM1*	XP_010649157.1	Vitvi04g01275	GSVIVT01018979001	4	18137893	18145402	2403	801
*VvIDN2a*	XP_002278500.1	Vitvi13g01987	GSVIVT01016528001	13	3094114	3102829	1926	642
*VvIDN2b*	XP_010651012.1	Vitvi06g00477	GSVIVT01024947001	6	5783783	5800541	1881	627
*VvIDN2c*	XP_002267670.1	Vitvi11g01117	GSVIVT01010892001	11	16213276	16217511	1740	579
*VvHEN1*	XP_002264328.3	Vitvi10g00954	GSVIVT01021670001	10	9034552	9050667	2796	932
*VvDCL1*	XP_010661522.1	Vitvi15g00864	GSVIVT01027462001	15	16551973	16563829	4425	1475
*VvDCL2*	XP_010649214.1	Vitvi04g01202	GSVIVT01019052001	4	17331795	17344656	4023	1340
*VvDCL3*	XP_010648400.1	Vitvi04g00186	GSVIVT01035494001	4	1758028	1786430	5067	1689
*VvDCL4*	XP_010656556.1	Vitvi11g00618	GSVIVT01001045001	11	6922239	7007216	4872	1624
*VvRDR1a*	XP_002284914.1	Vitvi01g00505	GSVIVT01011643001	1	5650814	5659360	2760	919
*VvRDR1b*	XP_002281315.1	Vitvi01g00503	GSVIVT01011645001	1	5627438	5633661	2832	943
*VvRDR2*	XP_002280099.1	Vitvi17g00776	GSVIVT01007792001	17	8977900	8986870	3384	1128
*VvRDR3*	XP_010656269.1	Vitvi11g00273	GSVIVT01015313001	11	2582031	2599309	2790	930
*VvRDR6*	XP_010648660.1	Vitvi04g00477	GSVIVT01035851001	4	4881183	4886034	3108	1035
*VvAGO1*	XP_002271225.1	Vitvi17g01218	GSVIVT01029383001	17	15920269	15928175	3117	1039
*VvAGO2*	XP_010655928.1	Vitvi10g01339	GSVIVT01026268001	10	15190286	15195417	2985	994
*VvAGO3a*	XP_003633060.1	Vitvi10g01346	GSVIVT01026261001	10	15100708	15106946	2952	983
*VvAGO3b*	XP_002274149.1	Vitvi10g01342	GSVIVT01026264001	10	15146900	15151225	2937	979
*VvAGO4a*	XP_002275928.1	Vitvi06g01020	GSVIVT01037488001	6	12830288	12839311	2742	913
*VvAGO4b*	XP_010653696.1	Vitvi08g00884	GSVIVT01025868001	8	10978235	10995752	2640	879
*VvAGO5a*	XP_010651834.1	Vitvi06g01378	GSVIVT01031430001	6	18665350	18674795	3099	1032
*VvAGO5b*	XP_010654011.1	Vitvi08g01559	GSVIVT01033726001	8	18308666	18314201	2556	852
*VvAGO6a*	XP_010657243.1	Vitvi12g00448	GSVIVT01030512001	12	6352396	6365926	2703	900
*VvAGO6b*	XP_019079568.1	Vitvi13g01085	GSVIVT01001941001	13	15074590	15079381	2646	882
*VvAGO7*	XP_002267746.1	Vitvi01g01134	GSVIVT01012490001	1	13829611	13833472	2670	889
*VvAGO10a*	XP_002279408.1	Vitvi05g00574	GSVIVT01018054001	5	5849436	5858977	2862	954
*VvAGO10b*	XP_010656388.1	Vitvi11g00408	GSVIVT01015464001	11	3938426	3949275	2718	906

### Phylogenetic and structural analysis

**Figure 1:**
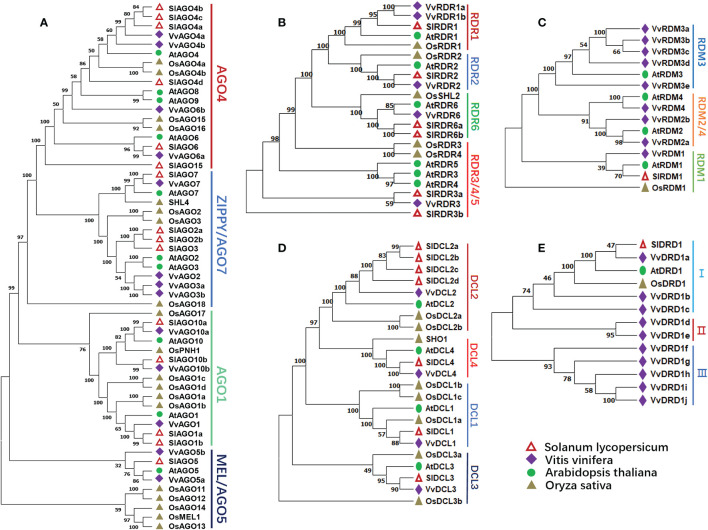
Phylogenetic analysis of AGO, RDR, RDM, DCL, and DRD proteins from grape, Arabidopsis, tomato, and rice. Hollow triangle, diamonds, circles, and triangle represent tomato, grapes, Arabidopsis, rice proteins, respectively. Numbers near the branches denote bootstrap values. **(A)** Phylogenetic tree of AGO proteins. AGO4, AGO7, AGO1, and AGO5 represent different subgroups. **(B)** Phylogenetic tree of RDR proteins. RDR1, 2, 3, 4, and 5 represent different subgroups. **(C)** Phylogenetic tree of RDM proteins. RDM1, 2, 3, and 4 represent different subgroups. **(D)** Phylogenetic tree of DCL proteins. DCL1, 2, 3, and 4 represent different subgroups. **(E)** Phylogenetic tree of DRD proteins. I, II, and III represent different subgroups.

To study the evolutionary relationship of the grape RdDM genes. The phylogenetic trees were constructed among Arabidopsis, grape, tomato, and rice RdDM subfamilies i.e., AGO, DCL, and RDR. According to **Figure 1A**, AGO proteins can be divided into 4 subgroups including AGO4, ZIPPY/AGO7, AGO1, and MEL/AGO5, and the protein affinities among dicots are closer. Further, AGO4 subgroup was more conserved with respect to proteins number. Except tomato all other have same number of proteins. The same trend of distribution in different subgroups was noted in all families of RdDM genes of grapes except RDM and DRD. Four VvDCLs proteins were divided in four subgroups: DCL1, DCL2, DCL3, and DCL4 (**Figure 1D**). Likewise, VvRDRs protein were distributed in RDR1, RDR2, RDR6, and RDR3/4/5 (**Figure 1B**). The VvRDMs and VvDRDs proteins of grapes were grouped into three sub-classes: RDM3, RDM2/4, RDM1 (**Figure 1C**) and I, II, III (**Figure 1E**), respectively. The more differences between grapes and Arabidopsis RDM and DRD protein numbers indicate that the RDM and DRD have observed more changes during the evolutionary process.

**Figure 2:**
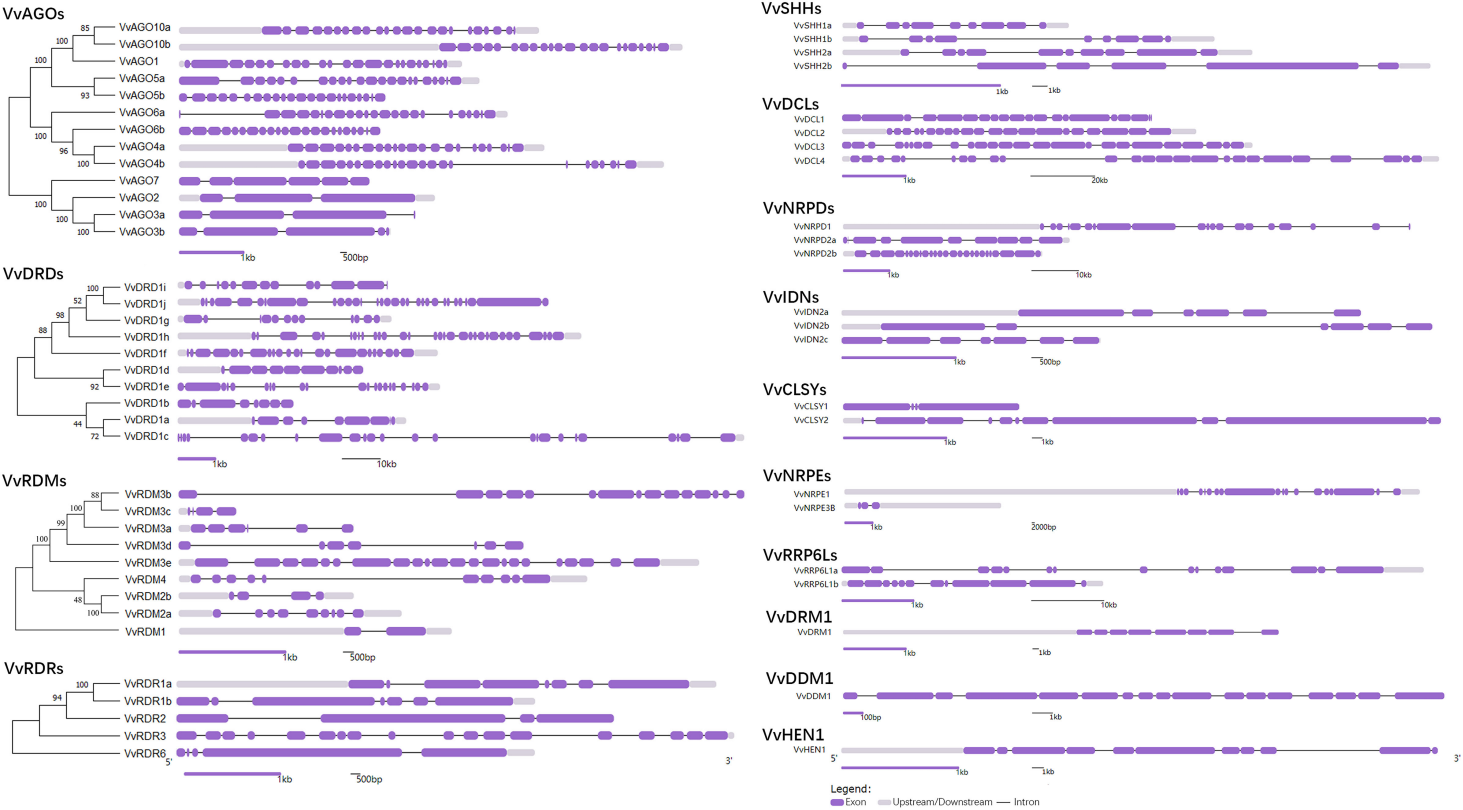
Phylogenetic tree and exon-intron distribution of *VvRdDM* genes. Purple boxes and black lines denote exons and intron, respectively.

For the better understanding of gene evolutionary process, the exon-intron structure of the grape RdDM genes was studied. The number of exons ranged from 2 (*VvRDM1*) to 32 (*VvDRD1j*) (**Figure 2**). As the AGO subfamily is concerned, there were almost the same number of exons within the same subgroup except AGO7 subgroup. This indicates that AGO7 is less conserved as compared to other groups. In addition, the DRD, RDM, RDR, SHH, DCL, NRPD, and IDN sub-family contained 8-32, 4-22, 4-18, 6-10, 21-25, 10-25, and 6-7 exons, respectively. Except, DCL and IDN sub-families, there were differences in the exon-intron distribution in the subfamilies and even within same subgroups, reflecting the diversity emerged during evolution. However, four duplicated gene pairs showed conserved (*VvAGO10a*-*VvAGO10b*, *VvIDN2a*-*VvIDN2b*, *VvAGO5a*-*VvAGO5b*, and *VvSHH1a*-*VvSHH1b*) exon-intron number.

**Figure 3:**
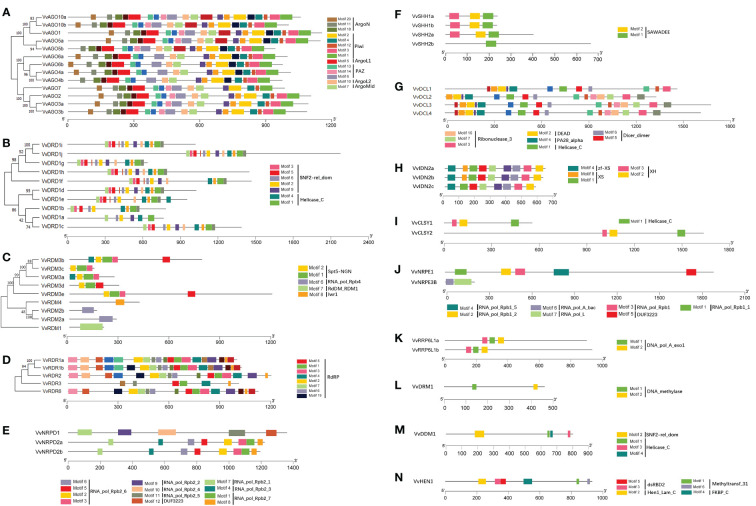
Conserved motifs analysis of *VvRdDM* genes. Different color boxes represent different motifs The letters **(A–N)** represent the different subfamilies.

Most of the subfamilies including AGO, DRD, RDR, SHH, DCL, IDN, RRP6L, and CLSY displayed conserved motif distribution patterns (**Figure 3**). Further, DRD, DCL, CLSY, and DDM subfamilies have a common structural domain (Helicase_C), but with different position and number. However, the members of RDM sub-family (supergene family) have different domains and protein sequences. Four duplicated gene pairs having same exon-intron distribution (as mentioned above) also showed similar motif distribution patterns.

### Promoter analysis of *VvRdDM* genes

The distribution patterns of different types of cis-regulatory elements may provide inklings about gene expressions and functions. A total of 18 cis-elements were identified and can be divided into 4 functional types; (i) Hormone responsive (MeJA, ABA, Auxin, SA, GA), (ii) Light responsive, (iii) Stress responsive (Defense and stress, Anaerobic, Drought, Cold), (iv) Participate in plant development (Endosperm, Meristem, Seed specific, Circadian control).

**Figure 4:**
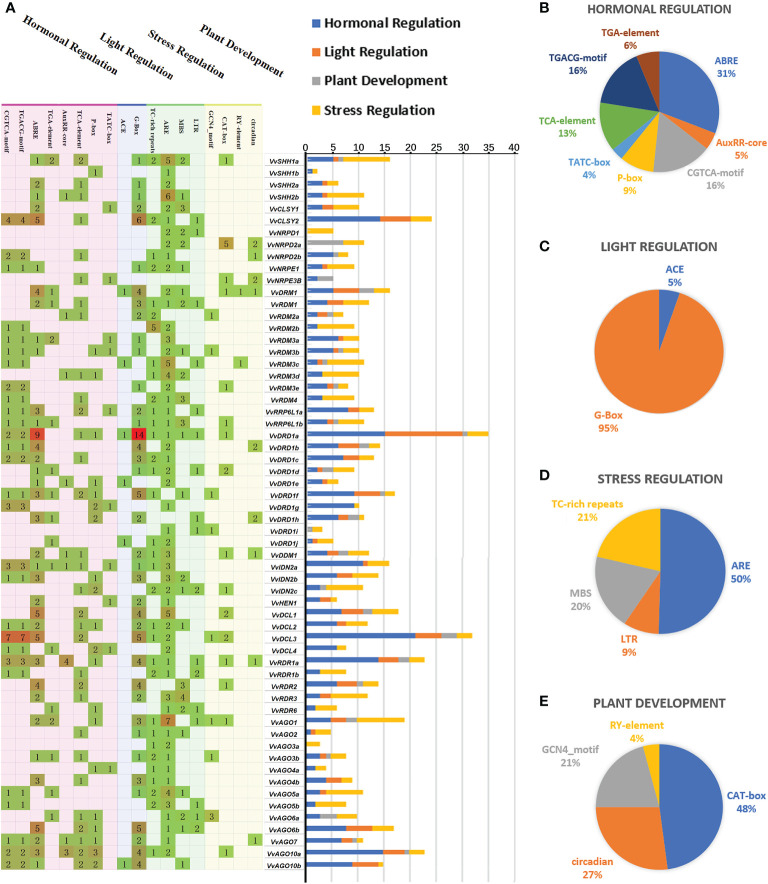
Cis-acting element analysis of *VvRdDM* genes. **(A)** The numbers and type of cis-elements in the VvRdDM promoter regions. The cis-elements belonging to different categorizes are denoted with different colors. **(B)** Relative percentage of hormonal regulation cis-elements. **(C)** Relative percentage of light regulating cis-elements. **(D)** Relative percentage of stress regulating cis-elements. **(E)** Relative percentage of plant development related.

Except four (*VvNRPD1*, *VvNRPD2a*, *VvDRD1i*, *VvAGO3a*), all other genes have hormone responsive elements including MeJA responsive element (CGTCA-motif, TGACG-motif), the ABA responsive element (ABRE), the Auxin responsive element (TGA-element, AuxRR-core), the SA responsive element (TCA-element) and GA responsive element (P-box,TATC-box) (**Figure 4A**). However, the ABRE were more abundant accounting up to 31% share in hormone responsive elements (**Figure 4B**).

Stress response cis-elements were present in most of the genes i.e., ARE (involvement in anaerobic induction), LTR (Low temperature-responsive elements), and TC-rich repeats (defense and stress response). Further, the presence of plant growth and development (CAT-box, circadian, GCN4-motif, and RY-elements) related elements suggests the involvement of *VvRdDM* genes in grapevine growth and development.

### Expansion patterns and chromosomal arrangements of *VvRdDM* genes

**Figure 5:**
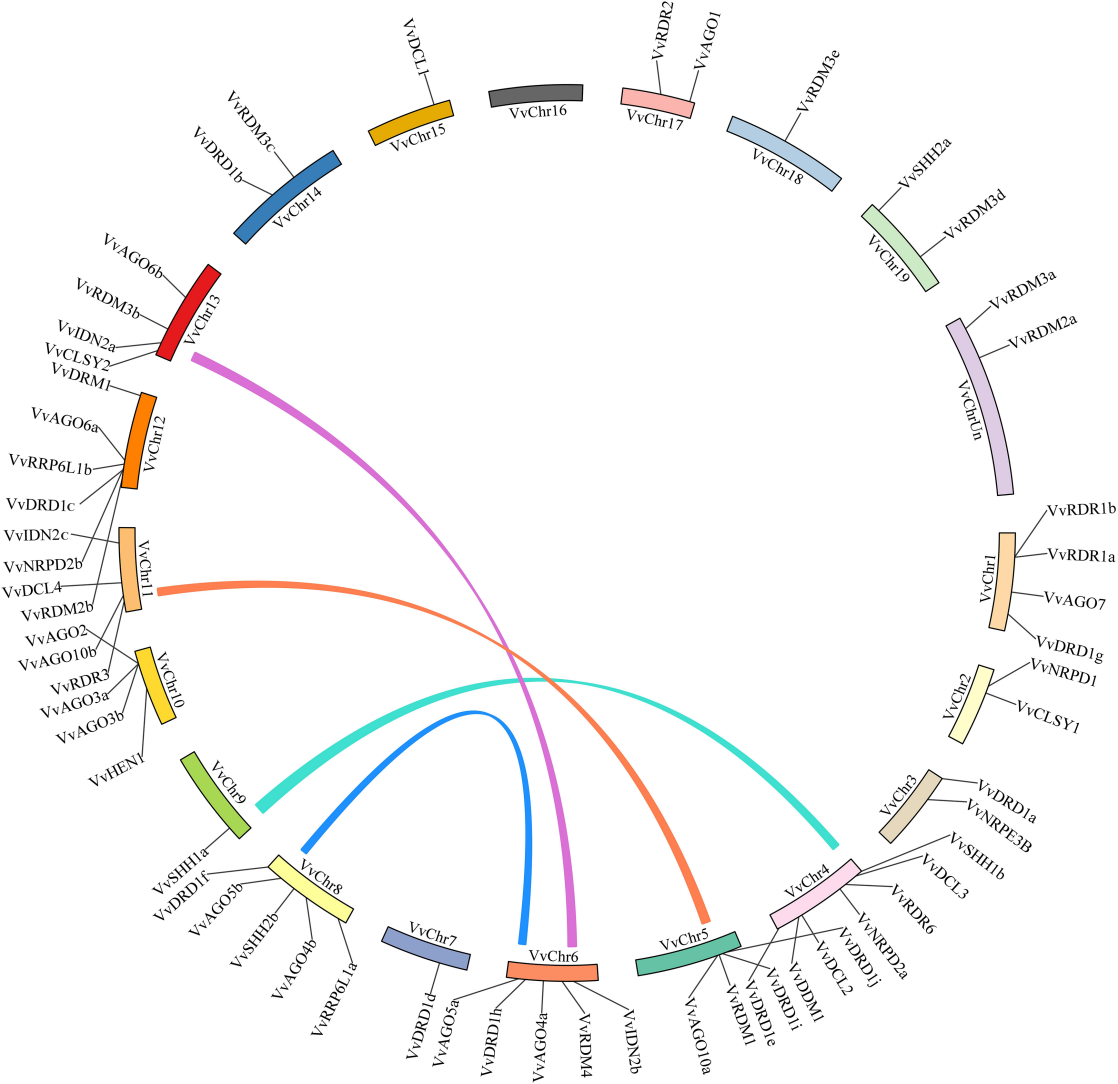
Chromosomal distribution and Synteny analysis. The corresponding genes on two chromosomes were regarded as segmental duplications and colored bars connecting two chromosomal regions indicate syntenic regions. Chr: chromosomes.

*VvRdDM* genes were mapped on grape chromosomes by utilizing the available grape genome annotation information. All genes were unequally present on 19 chromosomes and there was no gene on chromosome 16. Chromosome 4 contained the maximum number of genes (7) (**Figure 5**). Further, total 8 genes undergone segmental duplication in the form of 4 pairs (*VvAGO10b*-*VvAGO10a*, *VvIDN2a*-*VvIDN2b*, *VvAGO5a*-*VvAGO5b*, and *VvSHH1a*-*VvSHH1b*) and two events of tandem duplication (*VvRDR1a*-*VvRDR1b* and *VvAGO2*-*VvAGO3a*-*VvAGO3b*) were observed among five genes. Interesting, AGO family genes have undergone both duplication events and more than 50% genes (7 out of 13) are the result of duplication events. These results suggest that segmental and tandem duplication both have a role in the expansion of some *VvRdDM* sub-families. Further, Ka/Ks ratio was calculated for the estimation of selection pressure. According to the results all the duplicated genes have purifying mode of selection i.e., Ka/KS < 1 (**Supplementary Table S2**).

### Evolutionary relationships between arabidopsis and grapes

**Figure 6:**
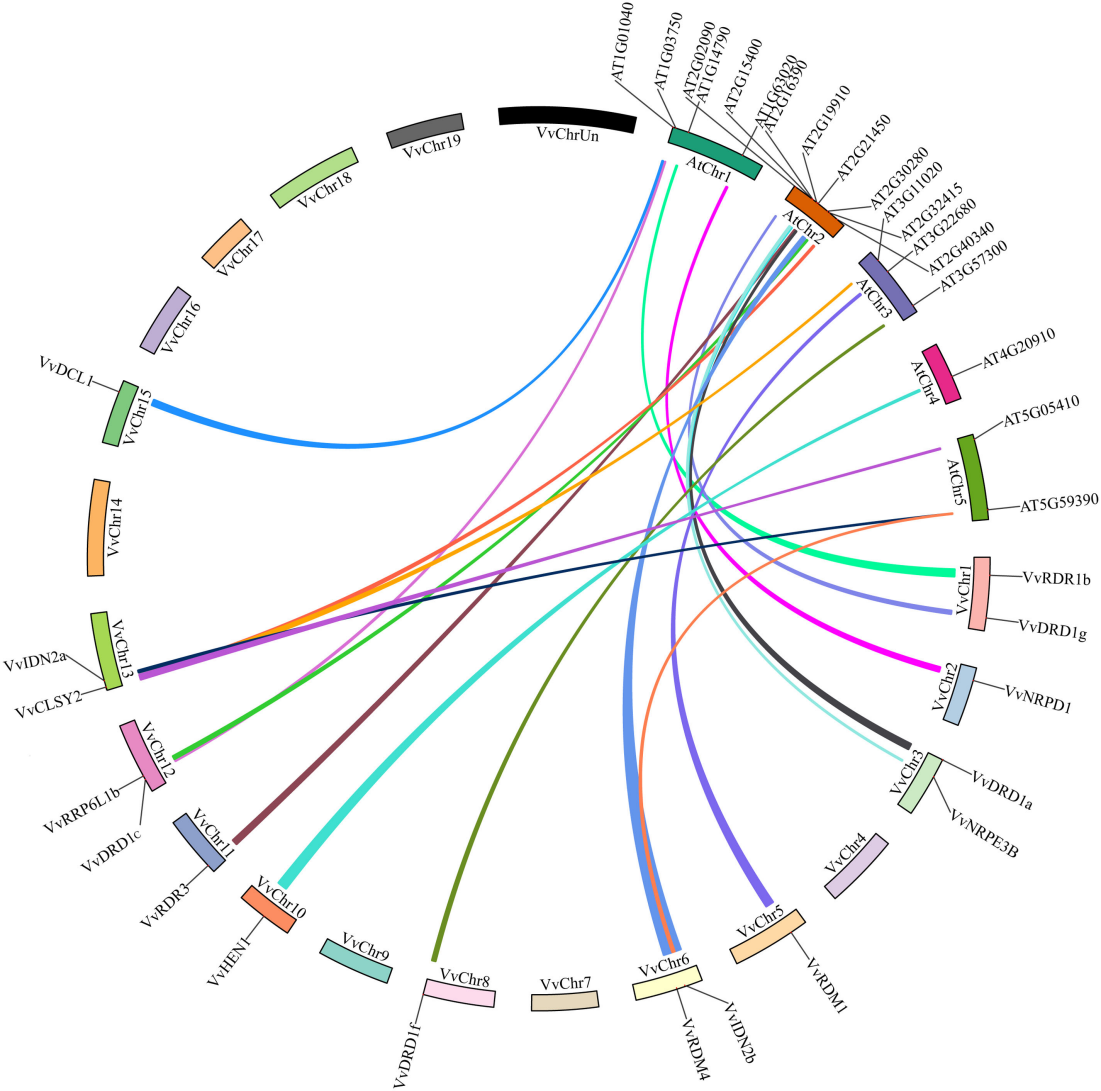
Synteny analysis of *VvRdDM* genes between grapes and Arabidopsis. Relative positions were estimated according to the grape and Arabidopsis chromosomes; colored lines denote syntenic regions.

To get further information about evolution of *VvRdDM* genes, the syntenic relationships between grape and Arabidopsis RdDM genes were found. There were 19 syntenic blocks among 17 *VvRdDM* and 19 *AtRdDM* genes (**Figure 6**; **Supplementary Table S3**). In each syntenic pair, both members belonged to the same subgroup. These syntenic relationships among grape and Arabidopsis suggest that these homologous genes have common ancestors before the speciation. Interestingly, *VvCLSY2* paired with three different Arabidopsis genes (*VvCLSY2*-*AT5G05410*/*AT3G11020*/*AT2G40340*). Further, *AT5G59390* also paired with more than one grapevine genes (*AT5G59390*-*VvIDN2a*/*VvIDN2b*). These results suggest that *VvCLSY2* and *AT5G59390* have gained more changes during the evolutionary process.

### Transcriptomic analysis of *VvRdDM* genes under biotic stress, abiotic stress and hormone treatment

**Figure 7:**
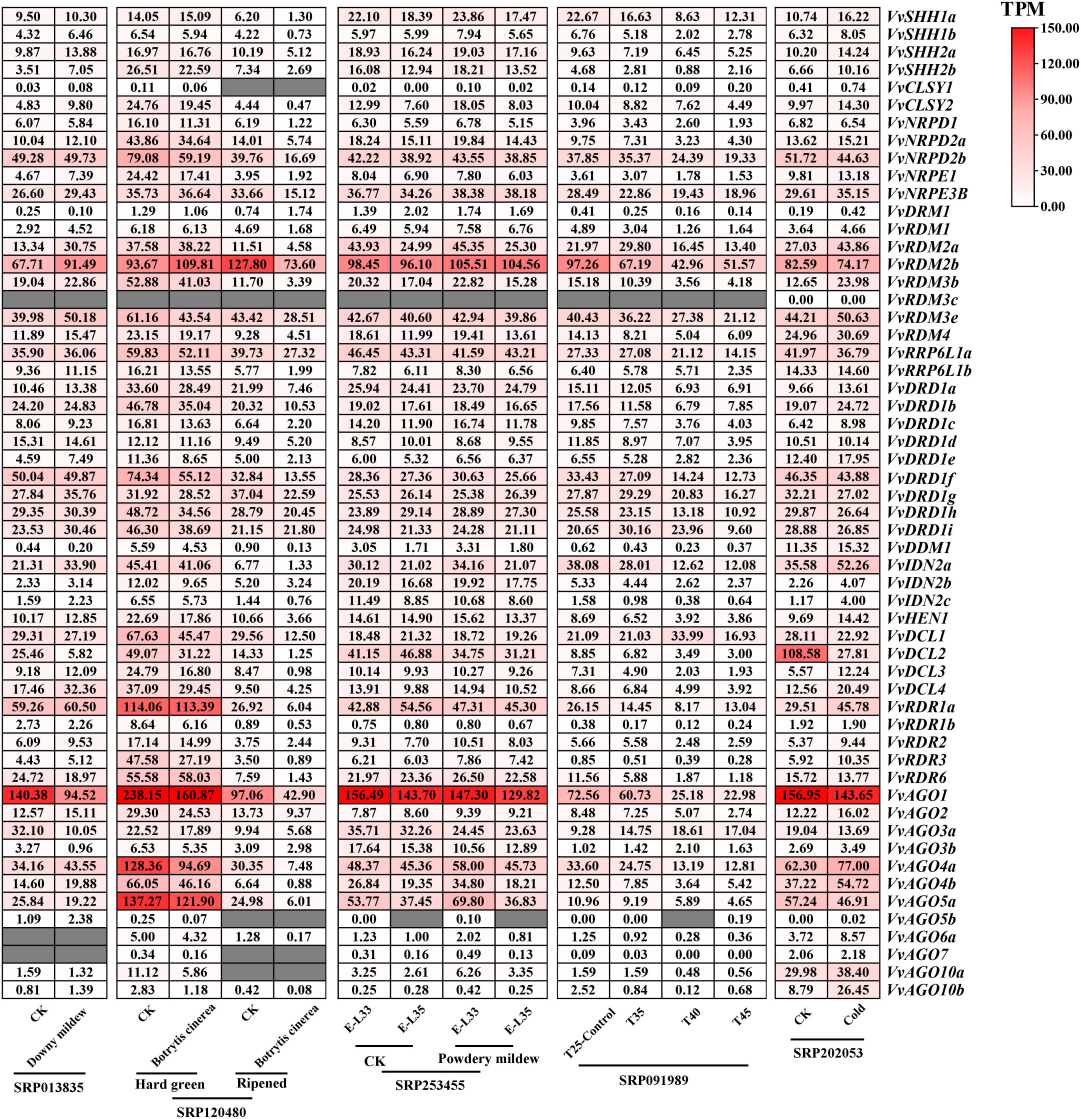
Transcriptomic expression of *VvRdDM* genes following biotic and abiotic stresses. Transcriptome data were downloaded from the NCBI SRA datasets (SRP013835, SRP120480, SRP253455, SRP091989 and SRP202053). For transcriptomic analysis plant samples following different stress were collected; For downy mildew infected leaves; Botrytis cinerea infected fruits at 2 stages (hard green and ripened stage); Powdery mildew treated fruits at 2 growth stages (EL33 and EL35). For high temperature stress, plants grown at 25°C were taken as control, and 35°C, 40°C, and 45°C were the treatments. Low temperature treatment is 4°C, plants grown at 25°C were taken as control. The white and red color scale denotes low and high expression levels, respectively. No gene expression is denoted with grey color. Numbers denote TPM of *VvRdDMs*.

To comprehensively explore potential functions of *VvRdDM* genes, expression patterns were investigated with a variety of transcriptome data. *VvRdDM* genes showed significantly differential expression against botrytis cinerea and downy mildew infection. For example, following *B. cinerea* infection, five genes (*VvRDR6*, *VvAGO4a*, *VvDCL3*, *VvRDM3b*, *VvNRPD1*) showed significant down-regulation and one gene (*VvDRM1*) displayed significant up-regulation expression at berry ripened stage. However, some of the *VvRdDM* genes (*VvRDR1a*, *VvAGO1*, *VvAGO4a*, *VvAGO5a*, etc) showed time specific expression under botrytis cinerea treatment i.e., higher expression level at berry hard green stage than ripened stage. Further, expressions of *VvDCL2*, *VvAGO3a*, and *VvAGO3b* were significantly down-regulated with downy mildew infection. However, none of the *VvRdDM* genes showed significantly different expression after infection with powdery mildew (**Figure 7**). After heat stress (35°C, 40°C, 45°C) treatments the number of significantly expressed genes was highest (13) at the 40°C. Interestingly, *VvAGO3a* showed a significant up-regulation against all temperature treatments.

Moreover, following cold treatment at 4°C, *VvIDN2c* and *VvAGO10b* were significantly up-regulated, and it is worth noting that the significantly down-regulated gene *VvDCL2* also had a high change in expression (108.58 to 27.81). *VvRdDM* genes showed better response to heat stress (**Figure 7**) i.e., *VvNRPD2b*, *VvRDM2b*, *VvIDN2a*, *VvAGO1*, and *VvAGO4a* showed decrease in expression with rise in temperature.

**Figure 8:**
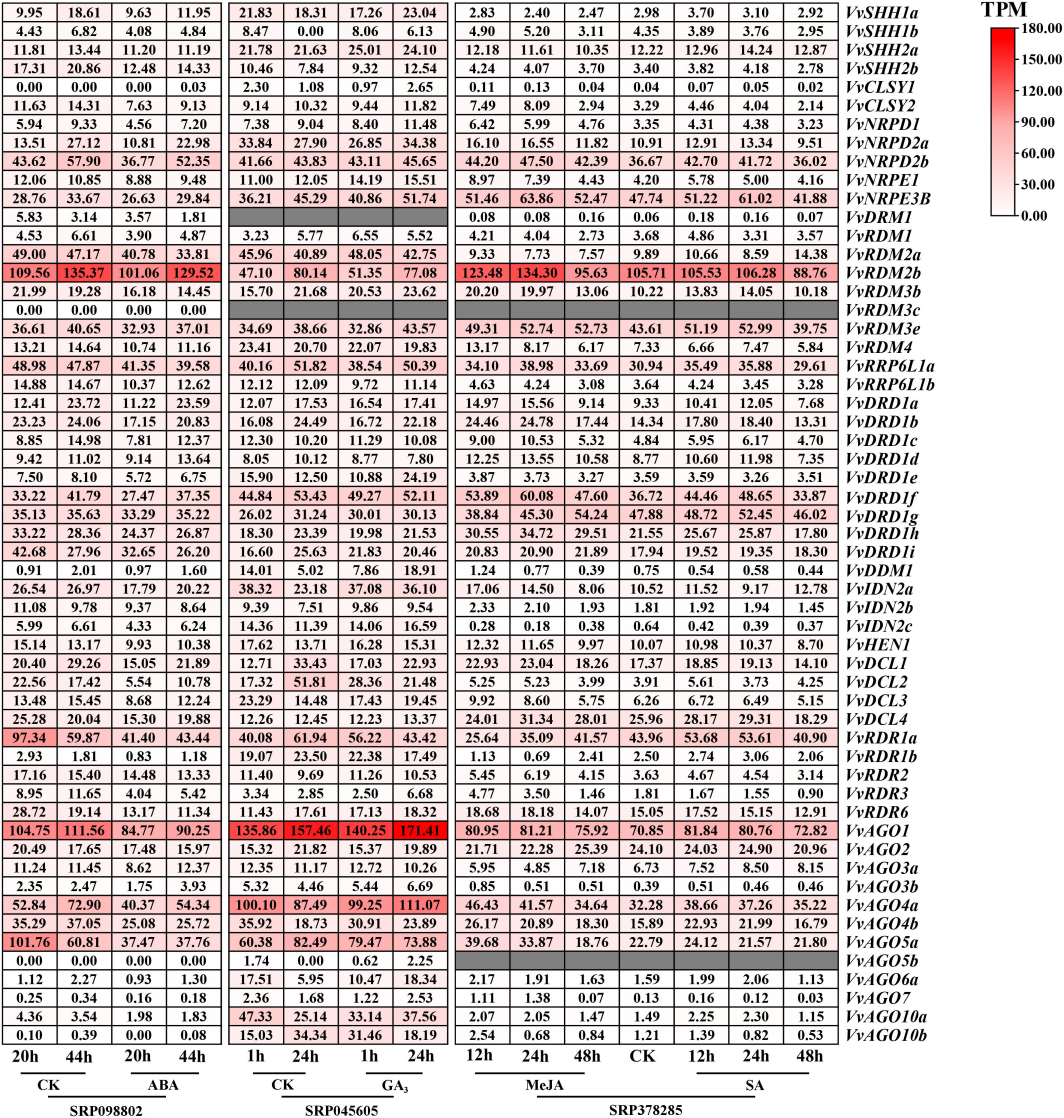
Transcriptomic expression of *VvRdDM* genes following different hormone treatments. Transcriptome data were retrieved from the NCBI SRA datasets (SRP09880, SRP045605 and SRP378285). The white and red color scale denotes low and high expression levels, respectively. No gene expression is denoted with grey color. Numbers denote TPM of *VvRdDMs*.

Under hormones treatment (**Figure 8**), most *VvRdDM* genes showed significantly different expression at 24 h post treatment. For example, *VvDDM1* and *VvAGO6a* were significantly up-regulated and *VvDCL2* was significantly down-regulated after GA_3_ treatment. Following MeJA treatment *VvRDR3* and *VvAGO7* were significantly up-regulated and *VvRDR1b* was significantly down-regulated. After ABA treatment some of the genes were significantly down-regulated i.e., *VvRDR1b*, *VvRDR1a*, *VvDCL2* and *VvAGO5a*.

### Expression profiling of *VvRdDM* genes in various organs and developmental stage

**Figure 9:**
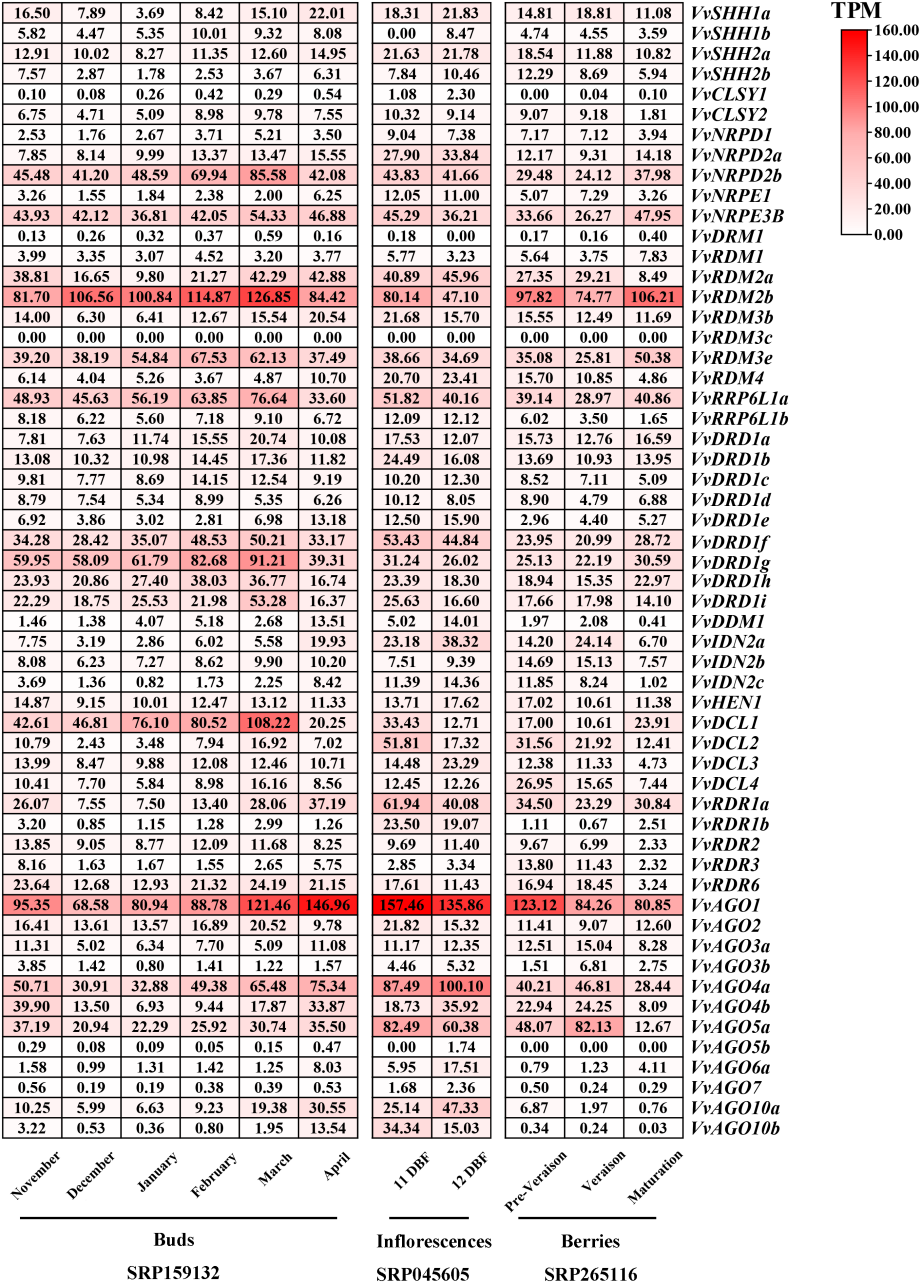
Transcriptomic expression of *VvRdDM* genes in different organs at various periods of time. The bud samples were collected at every month from November to April. The inflorescence samples were taken at 11 and 12 days before flowering (DBF). The fruit samples were collected at three developmental stages (pre-veraison, veraison, maturation). Transcriptome data were downloaded from the NCBI SRA datasets (SRP159132, SRP045605 and SRP265116). The white and red color scale denotes low and high expression levels, respectively. No gene expression is denoted with grey color. Numbers denote TPM of *VvRdDMs*.

Transcriptomic study of RdDM grapevine genes was undertaken to acquire some hints regarding the probable roles of RdDM grapevine genes in different organs (buds, inflorescences, berries, seeds). The results showed (**Figure 9**) that the *VvNRPD2b*, *VvNRPE3B*, *VvRDM2b*, *VvRDM3e*, *VvRRP6L1a*, *VvDRD1f*, *VvDRD1g*, *VvAGO1*, and *VvAGO4a* gene showed a broad spectrum of high expression in different organs at various stages, especially *VvRDM2b* and *VvAGO1*. During the process of bud development, *VvRdDM* genes (*VvNRPD2b*, *VvRDM2b*, *VvRRP6L1a*, *VvDCL1*, etc.) initially showed an increase in expression followed by a subsequent decrease. During April most of the genes showed a significant decrease in expression. However, some of the genes like *VvRDM2a*, *VvRDR1a*, and *VvAGO4a* showed a trend of initial decrease followed by increase in expression. Further, some genes (*VvRDM2b*, *VvDCL2*, *VvAGO5a*, etc.) also showed significant changes at the berry ripening stage, and this trend (change of expression) was also linked with the growth and development of grape organs.

**Figure 10:**
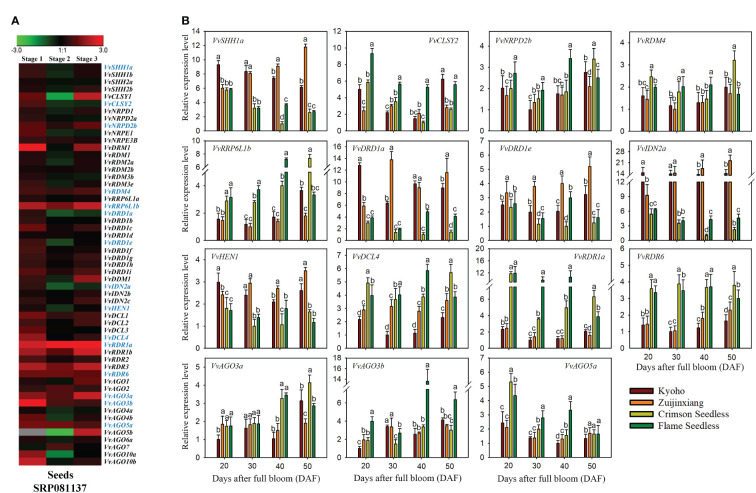
Transcriptomic and expression analysis of grapevine *VvRdDM* genes during seed development. **(A)** Heat map of grapevine *VvRdDM* genes in seeded and seedless progenies during seed development, transcriptome data were downloaded from the NCBI SRA datasets (SRP081137). Data were normalized by log_2_ the ratio of TPM (seedless/seeded) of each gene during the all three stages. The red and green boxes indicate the relatively high and low expression levels of each gene, respectively. **(B)** qRT-PCR analysis of *VvRdDM* genes in four cultivars during seed development. Different cultivars are denoted with different colors. Different letters denote statistically significant differences (ANOVA with a Tukey *post-hoc* analysis, 5% level). The mean ± S.D. of three biological replicates are mentioned.

The transcriptomic expressions of genes during seed development, are presented in the form of a ratio between seedless and seeded (Seedless/Seeded) cultivars at specific time points (initial stage, stage with the highest weight, and stage with the lowest weight during seedless progeny seed development) (Wang et al., 2016) (**Figure 10A**). The expression of *VvRDR1a*, *VvRDR1b*, *VvRDR3*, *VvRDR6*, and *VvRRP6L1b* was significantly higher in seedless progenies than in seeded progenies. Surprisingly, no gene showed significantly higher expression during all stages of seed development in seeded cultivar as compared to seedless. However, *VvAGO5b*, *VvAGO10a*, *VvCLSY1*, and *VvDRD1a* displayed significantly higher expression in seeded progeny only at one or at two times points. Overall, transcriptomic analysis suggests that some of the *VvRdDM* genes might have some roles in seed development or ovule abortion.

Based on transcriptomic analysis, 15 (*VvSHH1a*, *VvCLSY2*, *VvNRDP2b*, *VvRDM4*, *VvRRP6L1b*, *VvDRD1a*, *VvDRD1e*, *VvIDN2a*, *VvHEN1*, *VvDCL4*, *VvRDR1a*, *VvRDR6*, *VvAGO3a*, *VvAGO3b*, and *VvAGO5a*) genes were selected for further qRT-PCR analysis. The qRT-PCR analysis of selected genes was performed to check whether the trend of expression (differential) is common in different cultivars of grapes or it is variety specific. As shown in **Figure 10B**, *VvSHH1a*, *VvDRD1a*, and *VvIDN2a*, were significantly highly expressed during different stages of seed development in seeded progenies. Further all three genes showed relatively higher change ratio between seedless and seeded cultivars at 40 and 50 DAF as compared to earlier stages. Based on the findings, we postulate that these genes might have a significant role in seed development. On other hand, few genes were highly expressed in specific stages of seed development in seedless cultivars, for example, *VvRRP6L1b*, *VvDCL4*, *VvRDR1a*, and *VvRDR6*. However, *VvRDR1a* and *VvRDR6* showed significant higher expression during all phases of seed development in seedless cultivars than seeded ones. These findings imply that these genes may contribute to ovule abortion. The outcomes of qRT-PCR and transcriptome were generally in agreement.

## Discussion

In plants, RdDM pathway genes control the epigenetic states *via De novo* methylation and play important roles in multiple biological processes (Matzke and Mosher, 2014; Zhao and Chen, 2014; Matzke et al., 2015). Due to recent advancements in whole-genome mapping and next-generation sequencing, plant breeders are paying more attention in epigenetic research and its use in breeding programmes. The RdDM pathway genes have been characterized in many plants i.e., Arabidopsis, Citrus, Tomato, etc (Kurihara et al., 2008; Qian et al., 2011; Bai et al., 2012; Cao et al., 2016; An et al., 2017; Ahmad et al., 2019; Li et al., 2019; Gao et al., 2020; Mosharaf et al., 2020). However, there have been limited reports for RdDM pathway genes in grapes. Previously, Zhao et al. (2014) identified only three important RdDM pathway-related gene families in grapes i.e., AGO, DCL, and RDR. Here by using the latest high-quality grape reference genome data, we identified 60 RdDM grapevine genes belonging to 14 different subfamilies. Moreover, integrated bioinformatics, transcriptomics, and expression profiling to identify their potential functional roles.

Proteins sequences of one monocot (rice) and three dicots (Arabidopsis, grapes, and tomato) species were used to study the phylogenetic relationships of different RdDM families. Based on phylogenetic studies, VvAGO, VvDCL, and VvRDR proteins were more closely related with SlAGO, SlDCL, and SlRDR proteins, respectively. However, VvRDM proteins were closely related to AtRDM proteins. These results suggest that AGO, DCL, and RDR protein families of grapes and tomato have similarities in evolution and ancestral history. Likewise, RDM proteins of grapes and Arabidopsis might have common ancestors. The pattern of exon-intron distribution within a gene family can provide inklings about evolutionary history. Some of the *VvRdDM* genes families (AGO, DCL, and IDN) showed similar exon-intron numbers within the same sub-families, proposing that these subfamilies are functionally conserved. Other families showed differences in exon-intron number and distribution patterns even within the subfamilies. These results suggest that during the evolutionary process these subfamilies have either gained or lost exon/introns. Changes in exon number during the evolutionary process play important functions in the evolution of new gene families (Xu et al., 2012). Further, the newly evolved genes are initially redundant and gain new functions with the passage of time (Dias et al., 2003). Like intron-exon arrangement, most of the families (DRD, DCL, CLSY, and DDM) showed similar motif distribution patterns within the subfamilies suggesting these subfamilies are conserved during the evolutionary process. More interestingly, DRD, DCL, CLSY, and DDM all have a common domain (Helicase_C), which consists of ATP-dependent proteins which participate in epigenetic mechanisms *via* chromatin remodeling by using the energy provided by ATP (Flaus et al., 2006). This indicates this domain has some key roles in the RdDM pathways. However, RDM members showed differences in motif arrangements.

**Figure 11:**
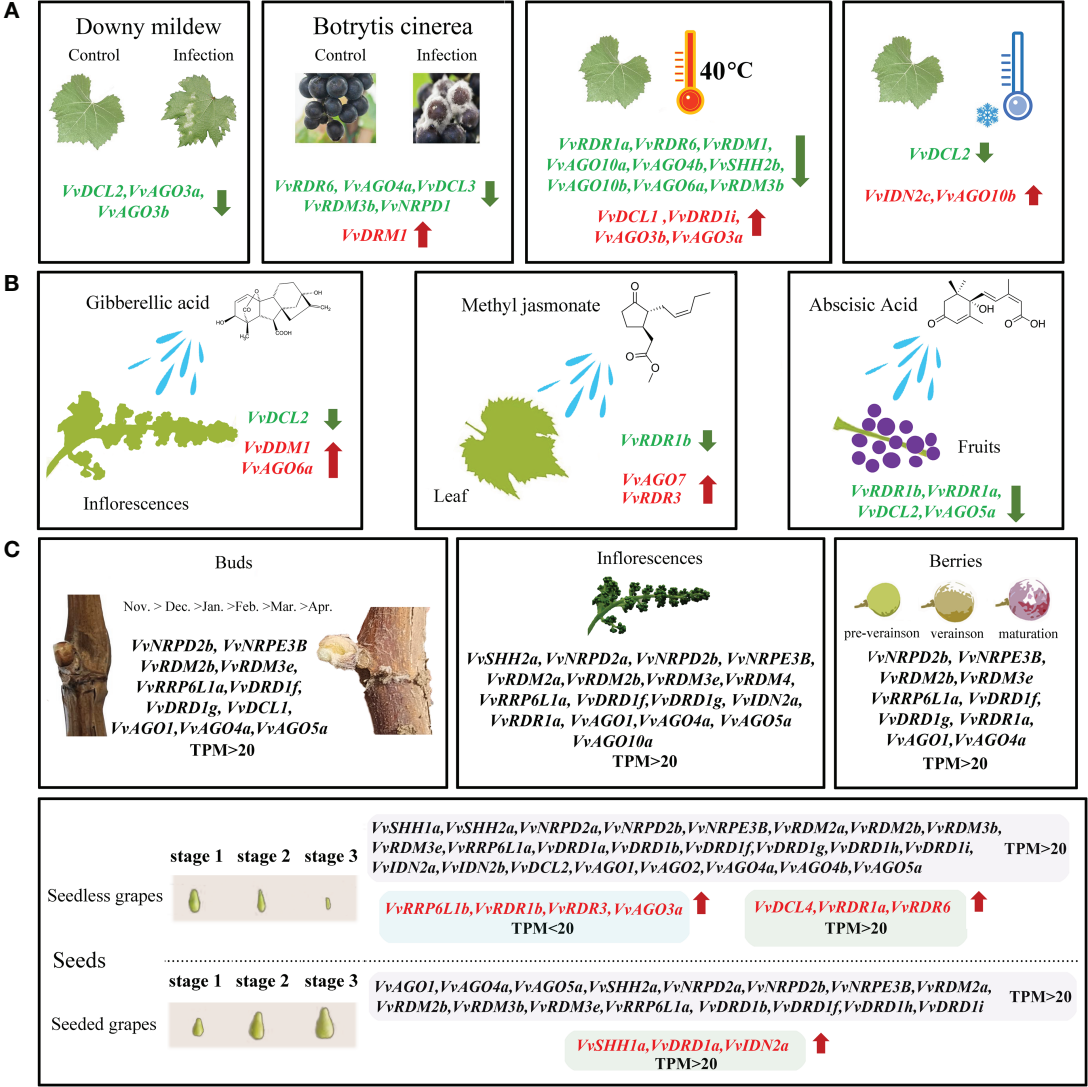
Transcriptomic and expression analysis of grapevine *VvRdDM* genes. The green and red font represents significant down and up-regulation, respectively. **(A)** The differential expression of *VvRdDM* genes following different biotic (downy mildew, botrytis cinerea), and abiotic (heat, cold) treatments. **(B)** The differential expression of *VvRdDM* genes under hormone treatments (GA_3_, MeJA, ABA). **(C)** The expression of *VvRdDM* genes in different organs: buds, inflorescence, seeds, and berries. The genes having TPM>20 are listed. In the seeds, the red font indicates that the expression was significantly up-regulated during the seed development in the seedless cultivars as compared with the seeded cultivars and vice versa.

Gene duplications and exon-intron patterns have played a key role in the expansion of gene families during the evolutionary process (Cannon et al., 2004). In case of duplicated gene pairs previous trend was observed i.e., members of four duplicated gene pairs (*VvAGO10a*-*VvAGO10b*, *VvIDN2a*-*VvIDN2b*, *VvAGO5a*-*VvAGO5b*, and *VvSHH1a*-*VvSHH1b*) showed a similar trend of exon-intron and motif distribution patterns. Moreover, some duplicated gene pairs (*VvAGO10a-VvAGO10b*, *VvAGO3a*-*VvAGO3b*) showed similar expression patterns under high temperature treatment (**Figure 11**). Only thirteen genes (8 segmental and 5 tandem) belonging to 4 families (AGO, IDN, SHH, and RDR) are the result of duplication events. All duplicated gene pairs have a purifying mode of selection. These results suggest that these four families are diversified and all others are conserved. Further, the AGO family is more diversified because 50% of members of this family have undergone duplication events. Overall, in the expansion of *VvRdDM* gene families, we noticed the previous trend that segmental duplication occurs more often than tandem duplication (Cannon et al., 2004). Synteny analysis can provide inklings about the gene functions. We identified 19 syntenic pairs between grape and Arabidopsis RdDM genes. These findings propose that these homologous genes have common ancestors before the speciation. However, relying solely on syntenic linkages to explain evolutionary relationships is difficult. Interestingly, two genes *VvCLSY2* and *AT5G59390* paired with more than one gene, implying that these genes have gained more changes during the evolutionary process. Based on the stated functions of orthologous genes in one species (Arabidopsis) functions can be predicted in other species (grapes).

According to promoter analysis, most of the *VvRdDM* genes have hormone-responsive cis-elements. However, ABA response element (ABRE) was a more abundant hormone-responsive cis-element, and genes *VvDCL2*, *VvRDR1a* (contained ABRE elements) significantly responded ABA treatment, moreover, the DEGs after MeJA treatment (*VvRDR1b*, *VvAGO7*-CGTCA-motif, TGACG-motif) and GA_3_ treatment (*VvDCL2*, *VvAGO6a*, *VvAGO7*-P-box) also contained related elements, suggesting the involvement of *VvRdDM* genes in hormone signaling pathways. Previously, Mosharaf et al. (2020) has also reported the presence of more hormone-responsive cis-elements in *CsRdDM* genes. Apart from hormone-responsive elements, stress and growth-related elements were also identified, signifying the role of RdDM genes in disease resistance and grapevine growth signaling pathways. These findings predict that regulation of RdDM genes is a complex process and rationalizes the need for further research.

Transcriptome data from SRA can provide some information for further research. In this study, previously published transcriptome data was used to evaluate the response and expression of *VvRdDM* genes against different treatments. Following different stress treatments or in different organs, the AGO family showed high expression or significant expression change as compared to control, suggesting it’s involvement in multiple biological processes (**Figure 11**). Previously, AGO family members have displayed diverse functions in different species. For example, in *A. thaliana* AGO mutant showed enhanced susceptibility to fungi (Ellendorff et al., 2009) and in another study *AtAGO4* mutants were associated with resistance to bacterial pathogens (Le et al., 2014). *SlAGO4A* has been reported for its role in salt and drought stress tolerance in tomato (Huang et al., 2016) and *OsAGO17* regulated seed size in rice (Zhong et al., 2020). AGO proteins are core components of the RNA-induced silencing complex (RISC) (Hutvagner and Simard, 2008), and this may be the potential explanation to its diverse roles. *VvRDM2b* and *VvAGO1* (**Figure 7** **–** **9**) gene showed a broad spectrum of high expression in different organs at various stages even under different treatment. *VvRdDM* genes not only showed diverse expression but also showed spatio-temporal expression patterns. For example, *VvRDR1a*, *VvIDN2a*, and *VvNRPD2b*, were significantly more expressed at the hard green stage than at the ripened stage. *VvAGO5a* was significantly differentially expressed at all stages of fruit ripening. Most of the *VvRdDM* genes showed significant down-regulation following different treatments (**Figure 11**). Surprisingly, *VvDCL2* displayed same expression trend under the conditions of downy mildew, low temperature, GA_3_ and ABA treatment. Further, the expression level of *VvDCL2* was significantly down-regulated following low temperature treatment (108 to 27) suggesting some role in the adaptation of grape to low temperatures. Our results are also supported by the earlier findings i.e., the activity of *DCL2* in *A. thaliana* was related with temperature (Zhang et al., 2012). *Prupe DCL* under drought treatment was differentially expressed in peach (Belal et al., 2022). These findings suggest, that the functions of DCL2 are diverse, and further research is needed for exploring the exact functions of *VvDCL2*. The *VvRdDM* genes showed differential expressions in different organs, indicating their involvement in the growth and development process. Our results are in line with the previous findings, as *AtRRP6L1* and *AtRRP6L2* (homologous genes of *VvRRP6L1a*) regulated flowering (Shin and Chekanova, 2014), *DDM1* (homolog *VvDDM1*) mutant effected seed development process (Xiao et al., 2006) in *A. thaliana*. Silencing of *FvAGO4* (homolog *VvAGO4a*) during fruit development resulted in early ripening of strawberry fruit (Cheng et al., 2018). Based on these findings, we speculate that *VvRdDM* genes can play critical role in the growth and development of grapes.

Expression patterns of fifteen genes during seed development were compared among four different (2 seeded and 2 seedless) cultivars of grapes. Interestingly, three genes (*VvSHH1a*, *VvDRD1a*, and *VvIDN2a*) showed higher expression in seeded cultivars especially at 40 and 50 DAF, signifying their key roles in grapevine seed development pathways. Previous results support our findings; for example, in *Hypericum perforatum* L, a gene with high similarity to *AtIDN2* demonstrated differential expression in the ovule of apomictic *H. perforatum*. Furthermore, in *A. thaliana*, silencing the IDN2 gene led to decreased seed set and changes in seed size (Basso et al., 2019). However, in seedless cultivars, *VvRDR1a* and *VvRDR6* showed significantly higher expression throughout all phases of seed formation. Similar trend of higher expression was noted during transcriptomic analysis. Relying on the findings, we can predict that these two genes might have roles in ovule abortion in grapes. Our results are in concordant with the previous findings of Grover et al. (2018) who noticed the abortion of *brassica rapa* seeds due to maternal alterations in the Pol IV-dependent small RNA pathway. They reported that RdDM pathway genes have critical roles in *brassica rapa* seed development. These findings rationalize the need for a future study about the functional characterization of these (*VvRDR1a* and *VvRDR6*) genes.

## Conclusion

A total of 60 RdDM genes in grapevine were identified, and comprehensive bioinformatic and expression analysis were performed. VvRdDM proteins were evaluated in terms of chromosomal positions, exon-intron distribution, phylogenetic, and evolutionary history. Expression analysis including transcriptomic analysis of *VvRdDM* genes following different stresses and hormonal treatments and qRT-PCR analysis of selected *VvRdDM* genes during different stages of seed development suggested their roles in multiple biological processes. *VvRDR1a* and *VvRDR6* gene were proposed as candidate gene resources for further functional characterization. Our study provides new resources for grape molecular breeding and inklings about the role of epigenetics in grapevine growth and development.

## Data availability statement

The datasets presented in this study can be found in online repositories. The names of the repository/repositories and accession number(s) can be found in the article/**Supplementary Material**.

## Author contributions

LW and RX designed the research. LW and GD supervised the experiments. RX performed most of the experiments. BA, LY and CL provided technical assistance. RX and XS analyzed the data. BA, RX and LW wrote the article with contributions of all the authors. All authors contributed to the article and approved the submitted version.
